# Preparation, Characterization, *In Vitro* Release and Degradation of Cathelicidin-BF-30-PLGA Microspheres

**DOI:** 10.1371/journal.pone.0100809

**Published:** 2014-06-25

**Authors:** Lili Li, Qifeng Wang, Hongli Li, Mingwei Yuan, Minglong Yuan

**Affiliations:** 1 Engineering Research Center of Biopolymer Functional Materials of Yunnan, Yunnan University of Nationalities, Kunming, Yunnan, China; 2 Department of Radiation Oncology, Sichuan Cancer Hospital, Chengdu, Sichuan, China; University of Akron, United States of America

## Abstract

Cathelicidin-BF-30 (BF-30), a water-soluble peptide isolated from the snake venom of Bungarus fasciatus containing 30 amino acid residues, was incorporated in poly(D,L-lactide-co-glycolide) (PLGA) 75∶25 microspheres (MS) prepared by a water in oil in water W/O/W emulsification solvent extraction method. The aim of this work was to investigate the stability of BF-30 after encapsulation. D-trehalose was used as an excipient to stabilize the peptide. The MS obtained were mostly under 2 µm in size and the encapsulation efficiency was 88.50±1.29%. The secondary structure of the peptide released *in vitro* was determined to be nearly the same as the native peptide using Circular Dichroism (CD). The ability of BF-30 to inhibit the growth of Escherichia coli was also maintained. The cellular relative growth and hemolysis rates were 92.16±3.55% and 3.52±0.45% respectively.

## Introduction

BF-30 is a 30-residue peptide isolated from the venom of the snake *Bungarus fasciatus*, which exhibits broad antimicrobial activity against bacteria and fungi. The peptide has strong antibacterial activity that has been identified *in vitro* against *Pseudomonas aeruginosain* in infected burns [1], [2]. BF-30 has a short onset of action when acting against P. aeruginosa and the drug-resistant bacteria, S. aureus. However, the peptide has a short half-life in serum [3], which may be caused by proteases present in serum [4]. Attempts have been made to increase the stability of BF-30 by chemical modification of the peptide through pegylation to extend the half-life [5], [6]. However, pegylation is a chemical modification method for increasing the stability of peptide by conjugating PEG to peptide or protein, the process may have an effect on the therapeutic effect of the peptide [3]. An alternative route to improve stability is to formulate the chemically intact peptide in a suitable carrier vehicle [3].

**Figure 1:**
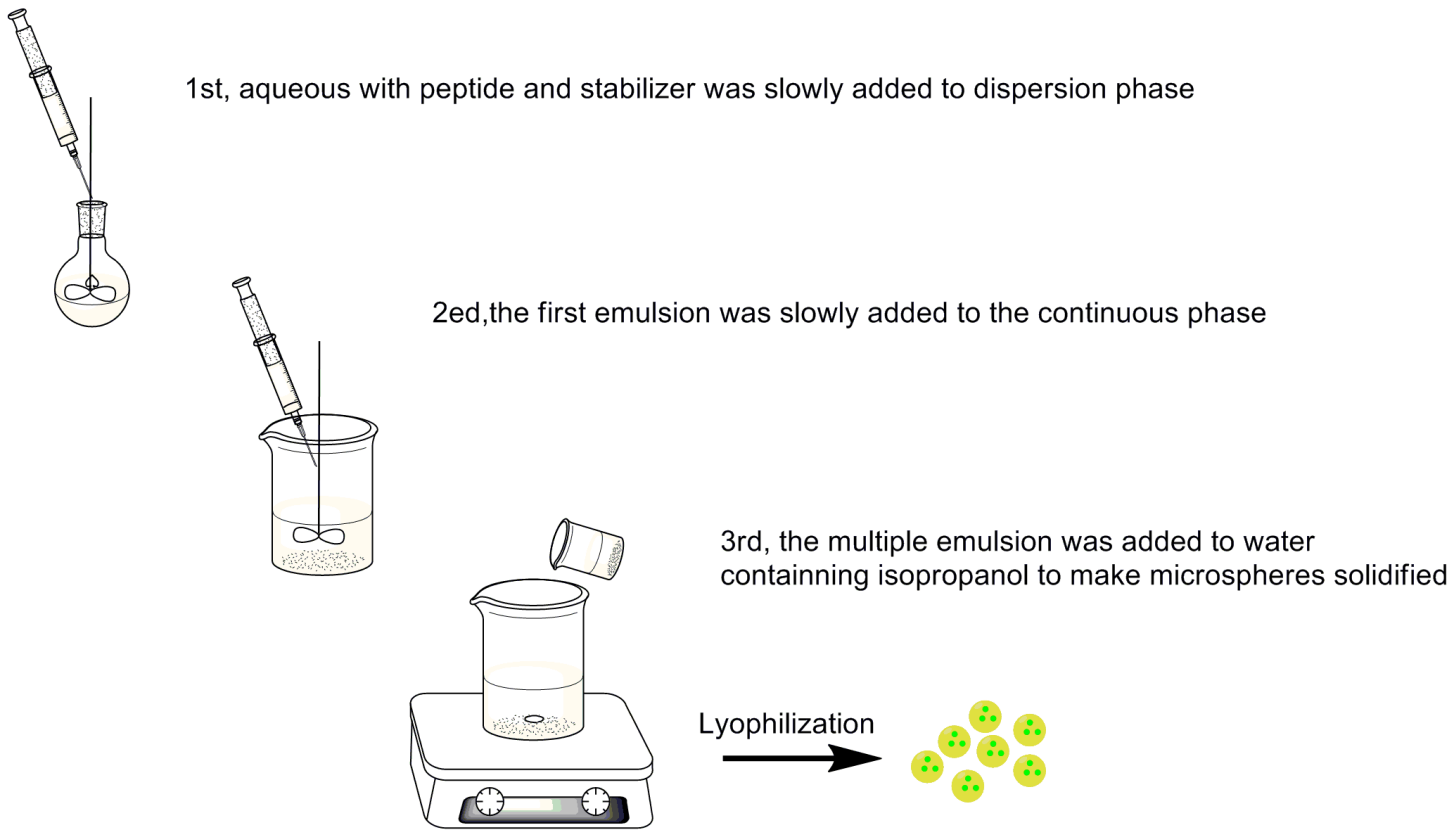
The process of preparing the microspheres.

Microsphere (MS)-based peptide polymer conjugation has proved to be a good delivery system for the encapsulation of other similar animal-based peptides [7], [8]. Hence this system was investigated as a potential method to encapsulate BF-30 for use in inhibiting the growth of bacteria. Figure 1 shows the preparation procedure of BF-30 loaded microspheres by W/O/W solvent extraction method, which containing twice emulsification and then solvent extraction by isopropanol solution.

Biomacromolecular therapeutics are a type of improved drugs that have been developed in recent years and biodegradable polymers such as polylactide have been used widely in protein and peptide delivery systems [7], [8], [9], [10]. As most biomolecular materials are hydrophilic, the MS are made by the water/oil/water emulsification solvent extraction method. In recent years, several kinds of novel polymers have been made to be used in drug delivery systems [11]. Of these polymers, poly(D,L-lactide-co-glycolide) (PLGA) has been in use the longest and has been approved by the US FDA for medical use [12]. In addition, MS containing luteinizing hormone-releasing hormone (LH-RH) formed using PLGA have been commercialized [9].

The aim of this research is to encapsulate BF-30 in PLGA MS, allowing a steady release of the peptide, while maintaining its activity.

## Materials and Methods

### Materials

PLGA 75∶25 with a weight average molecular mass (Mw) of 1.85×10^4^ Da and a polydispersity index of 1.30 was prepared through condensation polymerization in our lab. Polyvinyl Acetate (PVA, Mw = 75,000 Da, 88% alcoholysis, biochemical reagent) was purchased from Shanghai Jingchun Reagent Company Limited (Shanghai, China). Dichloromethane and isopropanol (analytical grade) were purchased from Tianjin Damao Chemical Reagent Factory (Tianjin, China). Sodium azide, trifluoroacetic acid, disodium hydrogen phosphate, monopotassium phosphate, and sodium chloride (analytical grade) were purchased from Chengdu Kelon Reagent Company (Chengdu, China). Acetonitrile (HPLC grade) was from MREDA Technology Inc (US). BF-30 (KFFRKLKKSVKKRAKEFFKKPRVIGVSIPF) with a Mw of 3637.50 Da was synthesized using solid phase synthesis by GL Biochem (Shanghai, China). Male rabbit (2.0 kg) was obtained from the Laboratory Animal Unit of Kunming Medical University (Kunming, China). Animal was anesthetized with pentobarbitone sodium (200 mg/kg,i.p.). Then drew blood from rabbit pericardium to heparinized tubes. All experiments about animals performed in this study were approved by the Committee on the Use of Live Animals in Teaching and Research of Yunnan University of Nationalities.

### Preparation of BF-30 loaded PLGA MS

BF-30 MS were prepared by dispersion followed by solvent extraction and evaporation according to the methods described in early articles by Ogawa and He [9], [13]. Briefly, 0.50 mL BF-30 solution in water (20 mg/mL) with excipient trehalose was slowly added to a solution of 5 mL of 16% (w/v) PLGA in dichloromethane (DCM) in an ice-bath under high-speed homogenization with Tween80 as surfactant. Then, the obtained emulsion was injected into a 1% PVA solution (the continuous phase) under the conditions described above. After the second emulsion process, the double emulsion was added to a 5% isopropanol solution to solidify the MS, then the MS were obtained by centrifugation for 10 min at 5000 rpm and washed with 0.30% mannitol/water solution three times.

### Surface morphology and size analysis

The surface morphology of the MS was analyzed using a JEM-100CX (HATACHI S-3000N, Japan) Scanning Electronic Microscope (SEM) and E-1010 Ion sputtering apparatus (HATACHI, Japan). Freeze-dried MS were placed on a metal stub, which was coated with conducting resin. The particle size and mean diameter distribution (polydispersity index) of the MS were measured by laser diffraction analysis using a Mastersizer 2000 (Malvern, UK). The preparation of the sample was as follows: the microparticles were dispersed in distilled water, then a dilute solution of uniformly dispersed microspheres were obtained by ultrasonic vibration. The samples were measured three times to calculate the mean diameter and polydispersity index (PDI).

### Encapsulation efficiency (EE) and microspheres yield rate

High encapsulation efficiency of MS was a key index of an excellent MS delivery system, and was the ratio between the amount of peptide entrapment and peptide added in the process of preparing MS. The amount of peptide entrapment was measured by dissolving 50 mg MS in 2.00 mL DCM (n = 3), then extracting the peptide twice with 2.0 mL Phosphate Buffer Solution containing Tween (PBST) (pH 7.40, 10 mM, containing 0.02% NaN_3_, 0.02% Tween80, and 150 mM NaCl) according to the method of OGAWA [9]. The concentration of peptide in the water phase was measured by HPLC, compared with a standard curve data of known concentrations of BF-30 solutions. In order to determine the amount of peptide loss during the inefficient extraction process, a certain amount (m_1_) of peptide solution was added into PLGA/DCM solution with the same concentration of MS preparing process. The BF-30 concentration in extraction liquid was measured in triplicate for three different batches, and different calibration values for inefficient extraction were detected for each sample as the method of Li [14]. The yield rate is another index of a delivery system which relates to the amount of microspheres obtained. The peptide entrapment efficiency was calculated according to the equation below.
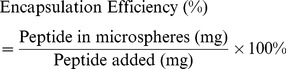
(1)

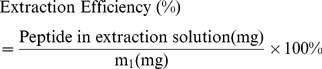
(2)

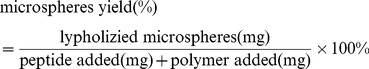
(3)


### *In vitro* release study

The MS loaded with BF-30 were incubated in a capped centrifugal tube containing 10 mL PBST (n = 3), kept at 37 °C and shaken at 120 rpm. At predetermined intervals, the tubes were removed and the samples were centrifuged at 3000 rpm for 10 min. Supernatant (1 mL) was extracted and an equal amount of fresh PBST was added. The peptide concentration in the supernatant was determined by the method described above. 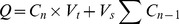
(4)


Q: cumulative release (µg)

C_n_: concentration of the release medium (µg/mL) at time t

V_t_:volume of the release medium, V_t_ = 10 mL

V_s_: volume of solution obtained from the release medium for testing, V_s_ = 1 mL

The release profile was obtained by plotting the cumulative release rate (Q/m_2_×100%) versus the release time.

Each experiment was repeated three times and the result is presented as the mean value of the three samples. The error bars in the plot show the standard deviation of the data.

### *In vitro* degradation study

The *in vitro* degradation study was similar to the release study, except that at the predetermined intervals, the degradation medium in the tubes was removed and the degraded MS were washed with distilled water to rinse off the buffer salt.

### HPLC and CD analysis

The concentration of the peptide extracted in the water phase was measured using reverse-phase HPLC (Agilent Technologies 1200 Series, Waters, xTerra, RP18, 5 µm, 4.6×250 mm column). The elution phase was A: water with 0.10% (v/v) trifluoroacetic acid and B: acetonitrile with 0.10% (v/v) trifluoroacetic acid; UV detection was at 220 nm [15]. The concentration of the acetonitrile phase was raised from 21.00% to 46.00% over 25 min using a linear gradient, and the sample size was 25 µL.

The peptide conformational change during the release period was measured on a far-UV (190–280 nm) Circular Dichroism Spectrometer Chirascan (Applied Photophysics Limited) at ambient temperature in a quartz cell (path length 0.05 cm) [16], [17] and compared with the native peptide.

### *In vitro* cytotoxicity analysis

The *in vitro* cytotoxicity of the peptide-loaded PLGA MS was characterized by the MTT (3-(4,5-dimethylthiazol-2-yl)-2,5-diphenyltetrazolium bromide) method, inendothelial cells at a concentration of 120 µg/mL (n = 5) [18], [19], under the conditions described by Shen [19]. The MTT method is based on the theory that succinate dehydrogenase in the mitochondria reduces dissolved MTT to a purple, water-insoluble formazan product in living cells, but not in dead cells. In detail, endothelial cells were grown at 37 °C under a 5% CO_2_ atmosphere in RPMI-1640 medium in a 96-well plate, supplemented with 10% calf serum in a fully humidified incubator. After 24 h, the culture medium was removed, MTT was added, and the samples were incubated for another 4 h. DMSO was added to dissolve the formazan crystals, and the optical density at 570 nm was measured on Multiskan Go spectrophotometer (Thermo, USA) with PBST as negative control. Each sample was prepared in triplicate. The cellular relative growth rate (RGR) indicating the ratio of living cells during the test was calculated according to the following equation:
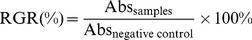
(5)


### *In vitro* hemolysis test

A hemolysis test was carried out to measure the ability of the biomaterial to destroy red blood cells when contacted with blood. The rate was quantified by measuring the suspension absorbance at 541 nm, which was consistent with the ferrohemoglobin released by destroyed red cells [20]. The hemolysis activity of BF-30-loaded PLGA MS was tested according to the method of Shen [19]. The hemolysis was quantified spectrophotometrically according to the method of Shen [19]and Fischer [16]. Blood obtained from rabbit pericardium was collected in heparinized tubes (n = 3), then centrifuged at 1500 rpm for 10 min on Eppendorf centrifuges (Germany, 5417R) to acquire rabbit red blood cells (RRBCs). Discarding the serum above by suction, the RRBCs were found deep in the centrifuge tube. Suspensions of the red blood cells were obtained by washing the RRBCs with a sterile physiological saline solution until the absorbance value of the positive control supernatant was located in the range 0.5–0.6 at 541 nm.

The hemolysis rate was calculated according to the following equation:
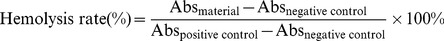
(6)


The sterile physiological saline solution and distilled water were the negative and positive controls respectively, and the corresponding hemolysis rates were 0% and 100% respectively.

### Biological activity

The ability of the released peptide to inhibit the growth of *Escherichia coli* was investigated according to the method of Kim [21]. The freeze-dried *Escherichia coli*, purchased from Guangdong Huankai Microbial SCI & Tech. Co. Ltd. (Cat. No. FSCC 149005, Lot. No. A0144B), were reconstituted in a brain-heart infusion broth. The bacterial solution was diluted to between 0.10 and 0.20 at OD625, an absorbance of half the McFarland standard. The absorbance value at 625 nm of the bacteria solution is proportional to the concentration of the bacteria in the solution [21]. Diluted bacterial solution (0.10 mL) was added to 96-well plates with 0.10 mL of the released peptide solution (n = 3), then incubated on a shaker at a constant temperature of 37°C and a rotation speed of 80 rpm. The absorbances at 625 nm were measured after 5 and 24 h. The percentage of bacterial inhibition was calculated according to the absorbance difference between the bacteria solution with and without the peptide after 5 and 24 h incubation using the equation:
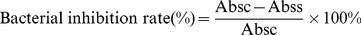
(7)


A_bsc_ and A_bss_ stand for the absorbance at 625 nm of the control group solution and the release medium with peptide, respectively.

## Results and Discussion

### Morphology characterization

**Figure 2:**
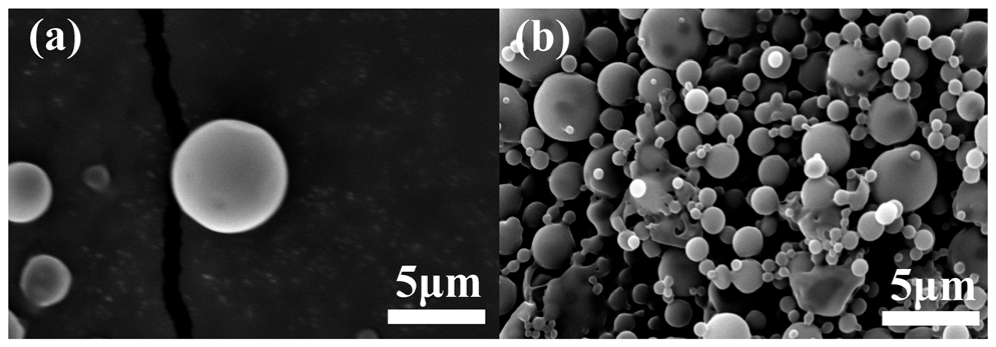
Morphology of peptide-loaded PLGA MS. (a) High magnification, (b) low magnification.

Microspheres prepared by the W/O/W emulsion/solvent evaporation/extraction method with a high yield of 76.64±8.07% according to the equation (3). SEM was used to investigate the morphology of the microspheres. Figure 2 shows the SEM images of lyophilized PLGA MS; most of the MS had spherical and smooth surfaces, but a few MS appeared pitted. As seen in Figure 2 (b), PLGA (75∶25) had little nonmicrosphere-forming ratio.

**Figure 3:**
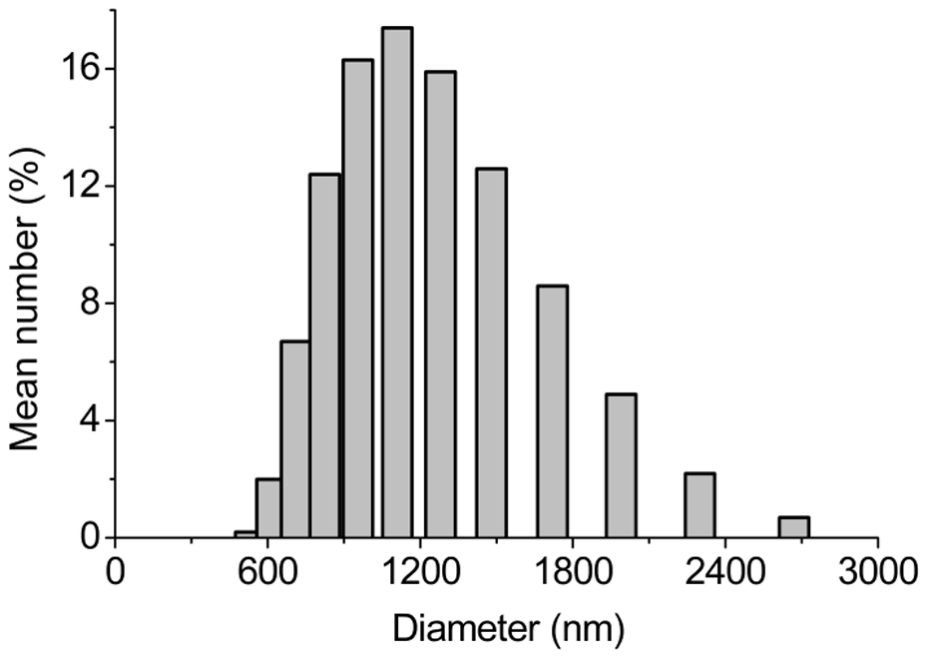
The size distribution histogram of the MS.

The MS size and size distribution were studied in a microsphere–water solution using Mastersizer 2000. As shown in Figure 3, the size of all microspheres obtained ranged from 500 to 3000 nm, with a mean diameter of 1460±14.02 nm. The majority of MS had a mean diameter between 800 and 1500 nm, in the submicro range [22], and a PDI of 0.24, which was<0.30. This means a narrow size distribution.MS with a diameter under 50 µm contribute to a steady release rate and decrease the burst release, thus the release kinetics would be close to a zero-order release mode [23], [24].

### Encapsulation and *in vitro* release of BF-30 loaded PLGA MS

**Figure 4:**
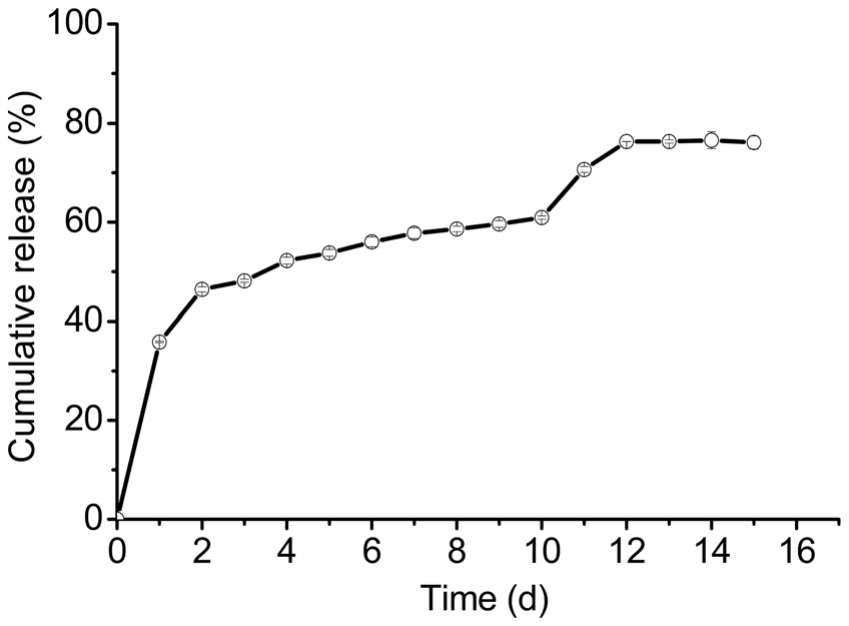
The release profile of the MS in PBST (pH = 7.40).

In our study, the concentration of the peptide had a liner range of 55.50 to 333 µg/mL, as measured by HPLC according to the standard curve equation: y = 4.88x–74.80, R^2^ = 0.9991; where y is the peak area and x is the concentration of the peptide (µg/mL). The encapsulation efficiency indicates the ratio of the actual amount of peptide encapsulated in the MS. In this study, the encapsulation efficiency was 88.50±1.29%, which was the calibration value according to inefficient extraction ratio 62.66±0.86%. Figure 4 shows the release profile of the BF-30 loaded PLGA MS. The release profile indicated the burst release was nearly 40% over the first release day, which may relate to peptide adsorbed on the surface of the MS [25], [26]. The peptide entrapped in the MS was released gradually with the degradation of polymer. The release process of the peptide containing three phases: the first burst release, lag and the second burst release. The second burst release often due to the erosion of polymer matrix [27]. At the end, 76.60±1.70% of the peptide encapsulated in the MS had been released.

### *In vitro* degradation and erosion of PLGA MS

**Figure 5:**
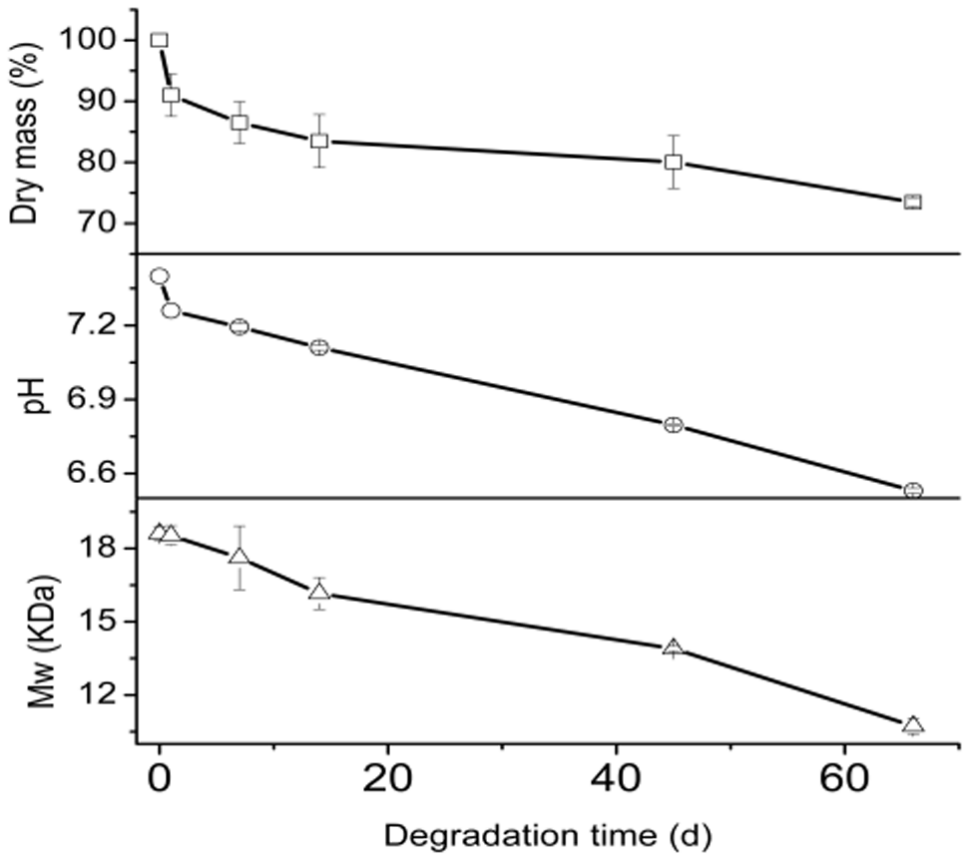
The degradation profiles of MS in PBST (pH = 7.40): pH, Mw, and dry mass – release time profiles.

Figure 5 shows a continued degradation of PLGA MS over 60 days with a significant drop in the pH value of the release medium and the dry mass (%). MS dry mass and Mw decreased during the first few degradation days were apparent, dry mass loss from 100% to 91% in the first day, the Mw of the BF-30 loaded microspheres dropped visibly from 18.50 to 17.60 KDa, which indicated that MS had undergone apparent degradation. As a result of acidic degradation products of PLGA-(lactic acid and glycolic acid) monomers and oligomers [24], pH value of the degradation medium dropped from 7.40 to 7.26, followed by a slower degradation process over the remaining 60 days.

### HPLC and CD analysis

**Figure 6:**
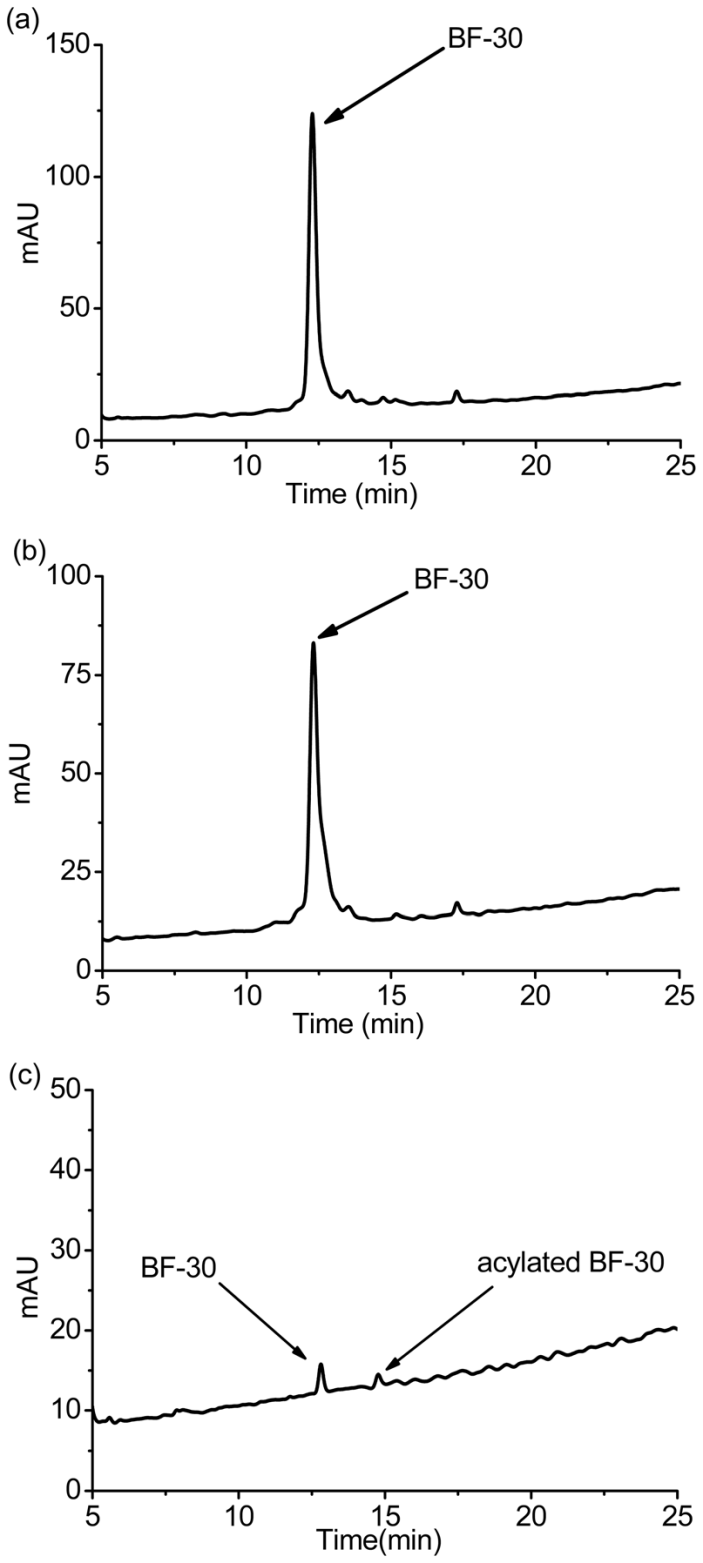
The elution curves of (a) the native BF-30 and (b) the peptide released on the 11th day, (c) the peptide released on the 10^th^ day.

As shown in Figure 6, the HPLC elution curve of the peptide released on the 11^th^ day(b) is similar to the native peptide(a), with the same retention time of 12.30 min. Curve (c) represents the peptide released in the 10^th^ day, which containing two peaks, peptide and acylated peptide. The HPLC results indicated that the peptide released on the 11^th^ day has the same polarity as the native peptide, but produced some acylated peptide on the 10^th^ day [28].

**Figure 7:**
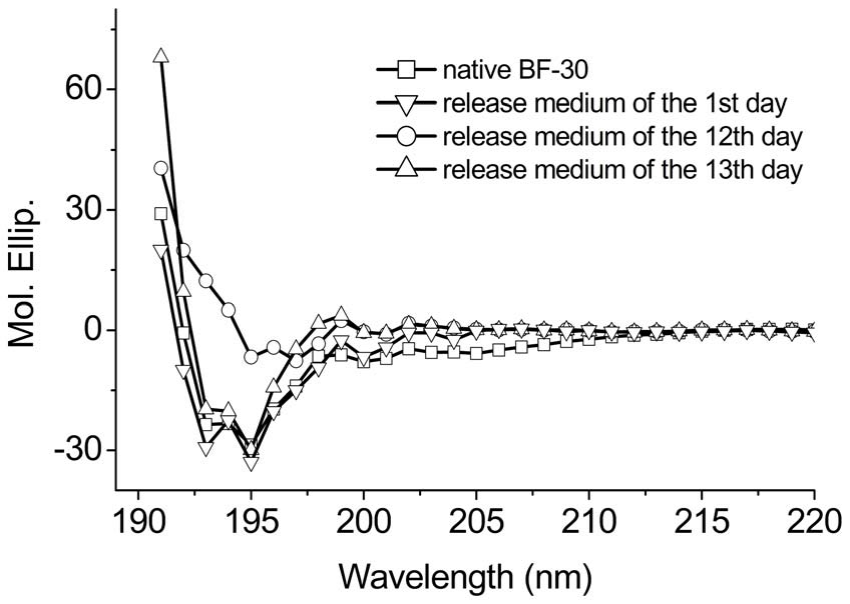
The CD spectra of the peptide released from the MS at different times compared with the native BF-30.

PLGA degraded to acidic compounds, including lactic and glycolic acids over time, which resulted in the observed decrease in the pH value of the degradation medium. Peptide would precipitate and assemble when the pH value of the medium is close to the isoelectric point. Fortunately, the isoelectric point of 11.79 for BF-30 is far above the observed pH value of the degradation medium. Thus the peptide in the MS would not precipitate and assemble and so most peptide encapsulated would be released [29]. Furthermore, Figure 7 shows that the far-UV CD spectrum of the peptide in the release medium of the 1^st^ day and of the later period of the release process, the 12^th^ and 13^th^ days, matching that of the native peptide. This demonstrates that the released BF-30 retains its α-helical secondary structure [30], indicating that the structure of BF-30 has not changed during the release process.

### Antimicrobial activity of the released peptide

**Figure 8:**
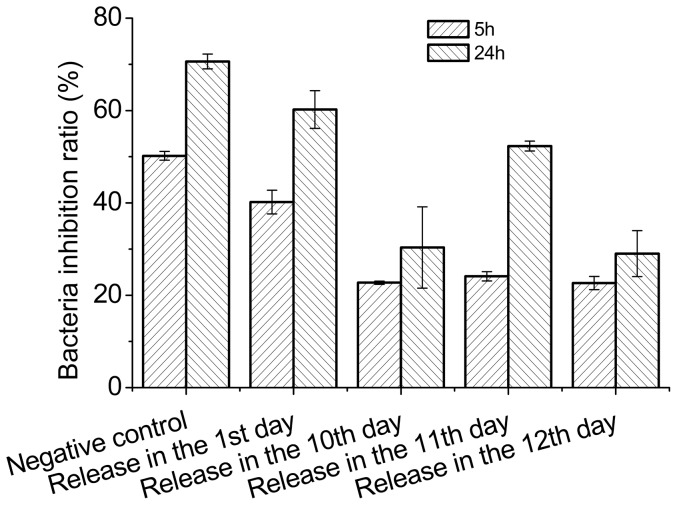
Antimicrobial activity against Escherichia coli of the native BF-30 and the peptide released from the microspheres on the 1^st^,10^th^, 11^th^, and 12^th^ days.

To test the biological effect of the released peptide, we investigated the activity of release medium on the 1^st^, 10^th^, 11^th^,and 12^th^ days against *Escherichia coli*, which is sensitive to BF-30 [1], [2]. As shown in Figure 8, the released medium of the 1^st^, 10^th^, 11^th^ and 12^th^ days were efficient in inhibiting the growth of *Escherichia coli*. The inhibition ratios and the peptide concentrations were 35.84±0.15%, 63.93±0.32%, 70.60±0.57%, 76.32±0.03%, and 25.50±0.34, 17.91±0.20, 19.70±0.10, 19.47±0.10 µg/mL on days 1, 10, 11, and 12 respectively, while the concentration of native peptide for antimicrobial test was 35 µg/mL. The inhibition ratio of the native peptide was higher than the 1^st^, 10^th^, 11^th^, 12^th^ days in all, and the 10^th^ day was lower than the 11^th^ and the 12^th^ day; this may be caused by a lower peptide concentration in the 10^th^ day, the acylated peptide, and the degradation of the MS, which makes the system unstable. We can see from Figure 6 that the 10^th^ day containing acylated peptide with lower concentration, and the 11^th^ day containing unbroken peptide compared with native peptide HPLC curves, but a little change in the 12^th^ day according to Figure 7. We can see from Figure 4 that the 11^th^ and the 12^th^ day were during the second burst release process. The peptide concentration in the release medium and the inhibition ratio on the 11^th^ day were higher than on the 10^th^ and 12^th^ days. Also, peptide in release medium of the 1^st^ day maintained its bioactivity according to Figure 8. The ability of the release medium to inhibit the growth of *Escherichia* was apparent after 24 h incubation with dilute bacteria solution. This means that the peptide has retained its bioactivity during the release period and the PLGA MS delivery system has the potential to protect the fragile peptide from adverse conditions.

### *In vitro* cytotoxicity analysis

A concentration of 120 µg/mL was used for cytotoxicity testing based on the amount of peptide encapsulated in the microspheres and the minimal inhibitory concentration (MIC) of 0.15 µg/mL for *Escherichia coli* [1].

**Figure 9:**
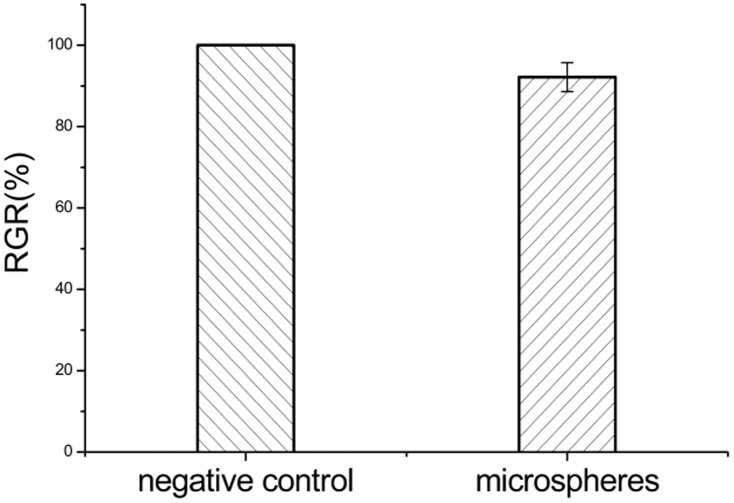
Cytotoxicity of the MS. PBST was used as the negative control.

After 48 h, the water-insoluble formazan formed by MTT in living cells was dissolved in dimethyl sulfoxide and the absorbance value of the samples at 570 nm was measured by absorbance spectroscopy on a Multiskan Go spectrophotometer (Thermo, USA). Figure 9 shows the results of the MTT assay, using PBST as the negative control. The BF-30-loaded microspheres showed 92.16±3.55% RGR, which indicated that no toxicity occurred at the concentration tested (120 µg/mL).

### *In vitro* hemolysis

**Figure 10:**
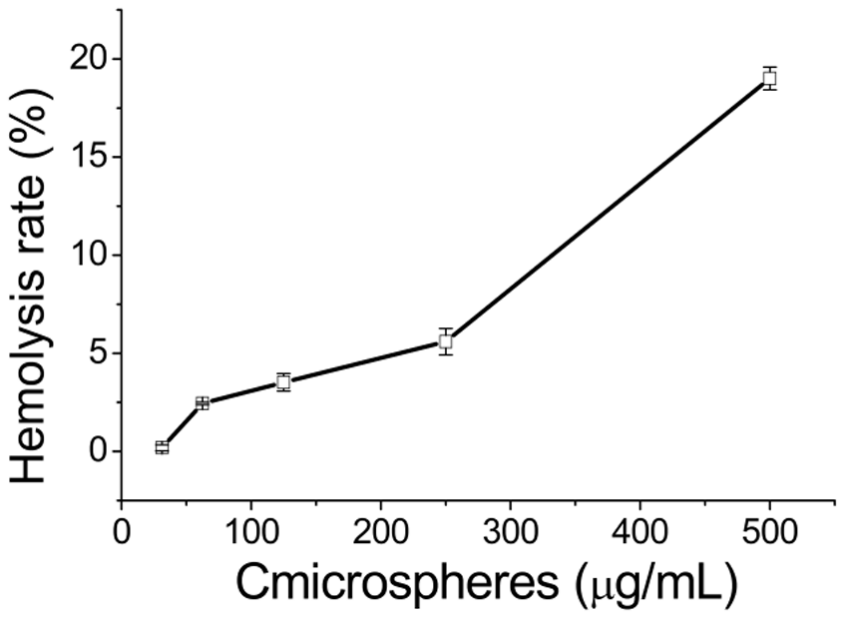
The hemolysis rate of BF-30-loaded PLGA MS at different concentrations.

Figure 10 shows the hemolysis level of microspheres at different concentrations. The OD value of the negative control, corresponding to 0% hemolysis, was 0.04±0.0003 and the value for the positive control, corresponding to 100% hemolysis, was 0.39±0.005. The hemolysis rate was 3.52±0.45%, which was lower than the ISO criterion of 5% [13], when the concentration of the microspheres was 125 µg/mL. These results indicate that the BF-30-loaded PLGA microspheres are safe when in contact with blood at the efficient concentration of 125 µg/mL.

## Conclusions

The antimicrobial peptide BF-30 of animal origin, which has antimicrobial activity, has been encapsulated into PLGA microspheres by the W/O/W double emulsion method with high encapsulation efficiency. The surface morphology of the MS was found to be spherical with a narrow size distribution. The delivery system released peptide over more than 15 days. The peptide retained its α-helix secondary structure during the release period. The BF-30 peptide released from the microspheres was found to be effective in inhibiting the growth of *Escherichia coli*, which means that the PLGA microsphere delivery system protected the peptide in terms of both structural stability and antimicrobial activity. In addition, the cytotoxicity and biocompatibility of the MS system was found to be within acceptable limits. In summary, the BF-30-PLGA microspheres hold great potential for use as an antimicrobial agent and the system may be useful for encapsulating other sensitive peptide-based drugs.
